# Comparison between a Novel Radiofrequency-Balloon and a Standard Cryo-Balloon in Pulmonary Vein Isolation: A Propensity-Score-Matched Analysis

**DOI:** 10.3390/jcm13040963

**Published:** 2024-02-08

**Authors:** Yannick Teumer, Clemens Miesbichler, Lyuboslav Katov, Benjamin Mayer, Wolfgang Rottbauer, Carlo Bothner, Karolina Weinmann

**Affiliations:** 1 Department of Cardiology, Ulm University Heart Center, Albert-Einstein-Allee 23, 89081 Ulm, Germany; yannick.teumer@uniklinik-ulm.de (Y.T.);; 2 Institute for Epidemiology and Medical Biometry, Ulm University, Schwabstraße 13, 89075 Ulm, Germany

**Keywords:** pulmonary vein isolation, atrial fibrillation, single-shot device, cryo-balloon, radiofrequency balloon

## Abstract

**Background/Objectives**: Single-shot devices are important tools for efficient pulmonary vein isolation (PVI) in atrial fibrillation (AF). In addition to the standard cryo-balloon (CB) catheter, a novel multi-electrode radiofrequency balloon-catheter (RFB, Heliostar, Biosense Webster, Irvine, CA, USA) with 3D-mapping-integration is available. Currently, there is no evidence allowing for a direct comparison between RFB-PVI and CB-PVI in a matched population. The study aimed to assess the procedural data, safety profiles, and outcomes of RFB-PVI versus CB-PVI. **Methods**: In this prospective registry study, symptomatic AF patients undergoing first-time PVI from January 2019 to April 2023, using RFB or CB, were included, with patients matched in a 1:2 ratio to reduce potential confounders. **Results**: The results from 171 consecutive RFB patients and 342 matched CB patients showed comparable recurrence-free survival after 12 months (81.3% RFB vs. 76.8% CB, *p* = 0.359). The RFB group had a longer procedure duration (88 vs. 73 min, *p* < 0.001) and longer fluoroscopy time (18.9 vs. 14.5 min, *p* < 0.001). **Conclusions**: In conclusion, the novel RFB system enables efficient and safe PVI, which is broadly comparable to the established CB system. However, the 3D-mapping integration in RFB did not reduce fluoroscopy time compared to CB.

## 1. Introduction

Atrial fibrillation (AF) is the most common type of arrhythmia, with a constantly growing incidence worldwide [1]. Ectopic beats from the pulmonary veins are considered the most common trigger of atrial fibrillation [2]. Therefore, pulmonary vein isolation (PVI) is recommended in the current atrial fibrillation guidelines of the American Heart Association and the European Society of Cardiology as a first-line therapy for symptomatic atrial fibrillation to reduce AF recurrence and quality of life by lowering the AF burden [1,3]. In symptomatic AF patients, PVI is especially superior to drug therapy regarding the prevention of atrial arrhythmia recurrence [4]. Single-shot devices are commonly used for PVI due to their procedural efficiency. In particular, the cryo-balloon (CB) system demonstrated a shorter procedure duration, fewer reconnected pulmonary veins in re-ablation procedures, and non-inferiority regarding atrial arrhythmia recurrence-free survival compared to the point-by-point radiofrequency (RF) PVI [5,6]. The lack of a 3D-mapping integration of the established single-shot devices results in higher radiation exposure compared to point-by-point RF PVI [5,7,8]. Combining the advantages of a single-shot device and 3D-mapping technology, a novel, compliant, multi-electrode RF-balloon catheter (RFB, Heliostar, Biosense Webster, Irvine, CA, USA) with full integration into 3D mapping technology (CARTO 3, Biosense Webster, Irvine, CA, USA) has emerged. The RFB comprises 10 gold-plated electrodes, and each electrode can deliver irrigated unipolar RF energy independently, providing circumferential, segmental, or even focal RF energy application to the pulmonary vein (PV) ostium. Several studies have demonstrated the feasibility and safety of the RFB system in establishing durable PVI [9,10,11,12].

To date, there has been no direct comparison of the novel RFB system and the established CB system in a matched population with paroxysmal and persistent AF. Our aim is to assess efficacy and safety by comparing the RFB and the CB systems.

## 2. Materials and Methods

### 2.1. Study Population

This prospective registry study included 513 patients with symptomatic paroxysmal or persistent AF who underwent a first-time PVI, either with the RFB (Heliostar, Biosense Webster, Irvine, CA, USA) or the CB (Arctic Front Advance Pro, Medtronic, Minneapolis, MN, USA), at our center (Ulm University Heart Center, Ulm, Germany). A total of 585 patients treated with the CB were screened between January 2019 and April 2023; 342 of them were enrolled according to the matching criteria. A total of 171 patients treated with the RFB were included between September 2021 and April 2023 due to catheter availability. Patients with a large left atrium (left atrial diameter > 55 mm), long-standing persistent AF, and age < 18 years were excluded. Data were prospectively collected in the ATRIUM registry (German Clinical Trials Register-ID: DRKS00013013). All patients provided written informed consent. This study was approved by the local ethics committee and was conducted in accordance with the Declaration of Helsinki.

### 2.2. Matching

**Figure 1:**
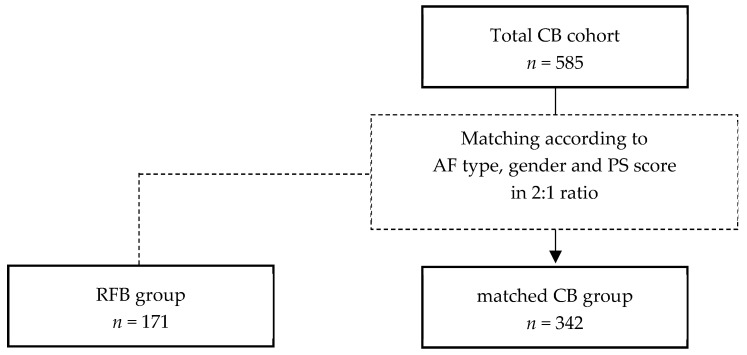
Depiction of the cryo-balloon control group’s matching to the radiofrequency-balloon group. AF, atrial fibrillation; CB, cryo-balloon; PS, propensity score; RFB, radiofrequency balloon.

Patients in the RFB group, who were described previously [12], were compared to patients of the CB control group following a hierarchical propensity score matching approach to reduce potential confounders [13]. Exact matching was performed for sex and atrial fibrillation type. Additional matching criteria, including age, left atrial diameter, body mass index, CHA_2_DS_2_-VASc-Score, and left ventricular ejection fraction, were considered for the propensity score distance. To enhance statistical power, an optimal matching strategy was utilized, matching RFB and CB control patients in a 1:2 ratio (Figure 1). Conducting a propensity score matching of the treatment and control groups lowers the risk of significant bias regarding the treatment effects compared to conventional regression models [14].

### 2.3. Ablation Procedure

CB-PVI and RFB-PVI procedures were performed under deep sedation. The CB-PVI sedation protocol included midazolam, fentanyl and propofol. Due to frequent pain-related excitation during RFB-PVI, a combination of midazolam, remifentanil, and propofol was used for RFB ablation [12]. Oral anticoagulation was not interrupted for the procedures. Antiarrhythmic drugs (AAD), in the sense of class I and class III antiarrhythmic drugs, were discontinued before the intervention. Beta-blockers were continued as part of the heart-failure therapy. Two venous punctures were performed in the right femoral vein. RFB-PVI punctures were guided by sonography. A steerable diagnostic decapolar catheter (Inquiry, 6F, Abbott, Chicago, IL, USA) was placed in the coronary sinus for electrogram recording. During ablation at the right-sided PVs, this catheter was temporarily positioned in the superior vena cava for phrenic nerve pacing. A single transseptal puncture was performed. The activated clotting time (ACT) was between 300 and 350 s during the procedure, using unfractionated heparin.

#### 2.3.1. Cryo-Balloon Ablation

**Figure 2:**
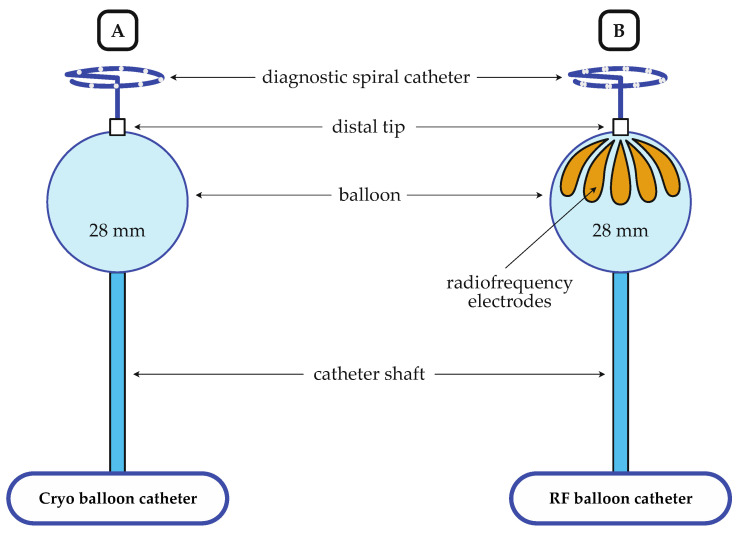
Schematic illustration of the cryo-balloon catheter (**A**) and the radiofrequency-balloon catheter (**B**); RF, radiofrequency.

After obtaining left atrial access, a steerable sheath (Flexcath Advance, Medtronic, Minneapolis, MN, USA) was advanced into the left atrium for CB-PVI. A 28 mm CB (Arctic Front Advance Pro, Medtronic, Minneapolis, MN, USA) with an inner lumen circular diagnostic catheter (Achieve, Medtronic, Minneapolis, MN, USA), which was advanced via the steerable sheath into the left atrium (Figure 2A and Table 1). PV occlusion was checked via the injection of a contrast agent. Ablation was performed according to a time-to-isolation (TTI) guided ablation protocol, as previously described by our group [15]. If TTI was between 30 and 60 s, a single 180 s freeze was delivered. If TTI was higher than 60 s, or TTI could not be observed, but PV isolation was achieved, a 180 s freeze was followed by a second 180 s freeze. If TTI was shorter than 30 s, freeze duration was decreased to 120 s.

#### 2.3.2. Radiofrequency-Balloon Ablation

The RFB ablation protocol was described in detail previously [12]. After obtaining left atrial access, a steerable sheath (Guidestar, 13.F, Biosense Webster, Irvine, CA, USA) and the RFB (Heliostar, Biosense Webster, Irvine, CA, USA, Figure 1B and Table 1) were advanced into the left atrium for RFB-PVI. An electroanatomic model (EAM) of the left atrium was created using a circular mapping catheter (Lasso NAV, Biosense Webster, Irvine, CA, USA) and a 3D-mapping system (Carto V7.2, Biosense Webster, Irvine, CA, USA). Subsequently, the RFB with an inner lumen circular diagnostic catheter (LassoStar, Biosense Webster, Irvine, CA, USA) was introduced via the steerable sheath into the left atrium. Due to the availability of the LassoStar NAV in August 2022, mapping was performed with the RFB and the inner lumen circular mapping catheter (LassoStar NAV, Biosense Webster, Irvine, CA, USA). To ensure optimal contact between the balloon and the tissue, a balloon inflation index > 0.8 and an impedance of 100 ± 20 Ohm were targeted across electrodes. Additionally, attention was paid to the anatomical coaxial alignment of the balloon and the PV guided by the electro-anatomical map and fluoroscopy. PV occlusion was determined by balloon electrode impedance and the injection of a contrast agent. After PV occlusion, two or three posterior-orientated balloon electrodes were selected with the help of the 3D map. Ablation was performed for 20 s at the posterior electrodes and for 60 s at the non-posterior balloon electrodes. RF energy (15 Watt) was temperature-controlled. The energy delivery was stopped earlier at the posterior electrodes if the esophageal temperature (ET) exceeded 39 °C. In cases where the posterior electrodes were already switched off and the ET continued to rise, the adjacent electrodes to the posterior electrodes were also switched off. The energy delivery was completely stopped if the ET exceeded 41 °C. Esophagogastroscopy (EGD) was scheduled within one week to detect thermal esophageal lesions (EDEL), and 40 mg pantoprazole per day was prescribed for 2 months if ET surpassed 42 °C. In the event of a detected EDEL, a Re-EGD was conducted two weeks later.

### 2.4. Phrenic Nerve Monitoring

While ablating the right-sided pulmonary veins in both CB-PVI and RFB-PVI, phrenic nerve pacing was performed. Furthermore, before delivering energy to the right superior pulmonary vein (RSPV), phrenic nerve pacing was conducted using the anterior-oriented RFB electrodes at maximum output to assess the proximity of the phrenic nerve to the ablation site. If phrenic nerve capture occurred, adjustments were made, such as repositioning the balloon or deactivating single balloon electrodes, as part of a segmental energy delivery strategy.

### 2.5. Periprocedural Management and Follow-Up

All individuals underwent continuous monitoring for a duration of 24 h post-procedure, encompassing clinical examination, transthoracic echocardiography, and a 12-lead resting electrocardiogram (ECG). Following pulmonary vein isolation (PVI), follow-up appointments at the outpatient clinic were scheduled at 3, 6, 12, and 24 months post-procedure. In the event of symptomatic recurrence, evaluations, including clinical examination, 12-lead resting ECG, and a 7-day Holter ECG, were conducted. Atrial arrhythmia (AT/AF) recurrence was defined as any atrial tachyarrhythmia lasting longer than 30 s after a 3-month blanking period.

### 2.6. Study Endpoints

The primary endpoint was defined as AT/AF recurrence-free survival after 12 months according to Kaplan–Meier estimation in the RFB group in comparison to the CB group. Secondary endpoints were total procedural duration, fluoroscopy time, left atrial dwell time, single-shot isolation rate, time-to-isolation, and the energy applications required per vein to succeed in isolation. Time-to-isolation was defined as the time from the beginning of the energy application until PVI was achieved. Procedural duration was defined as the time from the femoral puncture to catheter removal.

Primary safety endpoint was defined as a composite of fatality and any major periprocedural complication. Major periprocedural complication was defined as pericardial effusion with the need for pericardiocentesis, transient ischemic attack (TIA), stroke, persistent phrenic nerve palsy (PNP), atrio-esophageal fistula, and vascular access complications requiring specific treatment. Secondary safety endpoints were defined as pericardial effusion without the need for pericardiocentesis, transient PNP, and vascular access complications, which do not require specific treatment.

### 2.7. Statistical Analysis

For statistical analysis, SPSS Statistics (V29, IBM, Armonk, NY, USA) was utilized. Categorical variables were presented as frequencies and assessed using the Chi-square test or Fisher’s exact test, as appropriate. The normal distribution of numeric variables was assessed via the Shapiro–Wilk test, and equal variance was examined using Levene’s test. Normally distributed variables were expressed as mean ± standard deviation and analyzed using Student’s *t*-test. Non-normally distributed variables were described as median with interquartile range (IQR) and analyzed with the Mann–Whitney rank sum test. Due to the matched design of this study, the outcome parameters of primary interest were analyzed using approaches which can deal with correlated data. Consequently, a linear mixed model was applied to analyze continuous outcomes, a conditional logistic regression was applied for dichotomous outcomes, and recurrence-free survival was analyzed using Kaplan–Meier analysis and a Cox proportional hazards regression model using a robust sandwich covariance estimator. A *p*-value < 0.05 was considered significant.

## 3. Results

### 3.1. Baseline Characteristics

In the RFB group, 171 consecutive patients (36.8% female) were included. The CB group consisted of 342 patients (36.8% female), who were matched to the RFB group, out of a total of 585 patients treated with the CB system (Figure 2). Detailed patient characteristics are summarized for both groups in Table 2. There were no significant difference between groups regarding the baseline characteristics.

### 3.2. Procedural Characteristics and Ablation Data

In the RFB group, the median procedural duration was 88 (70–115) minutes and median fluoroscopy time was 18.9 (13.9–29.6) minutes (*p* < 0.001). In comparison, the median procedural duration (73 (54–97) min) and median fluoroscopy time (14.5 (9.8–21.4) min) were significantly shorter in the CB group (*p* < 0.001). The median left atrial dwell time was significantly shorter in the RFB group (23 (15–36) versus 28 (20–41) min; *p* = 0.006). Procedural characteristics are summarized in Table 3.

The treated PVs are depicted for both groups in Table 4. Except for the right middle pulmonary vein (RMPV), time to isolation was significantly shorter in the RFB group. the single-shot isolation rate showed no difference in both groups for the left superior PV (LSPV), the right superior PV (RSPV), and the right inferior PV (RIPV). At the left inferior PV (LIPV), the single-shot isolation rate was significantly lower in the RFB group (*p* < 0.001).

### 3.3. Safety

No fatality occurred in either group. In the RFB group, no major complications were detected. In the CB group, one TIA with complete convalescence, one pericardial tamponade that required pericardiocentesis, and two persistent PNPs occurred. Concerning minor complications, one transient PNP was detected in the RFB group, which resolved within 48 h. In the CB group, three vascular access complications (one femoral pseudoaneurysm, two intramuscular hematomas) occurred; all of them were treated conservatively. Furthermore, four transient PNPs were detected in the CB group. There were no significant differences regarding the overall PNP rate between both groups. An EGD was conducted in 35 RFB patients who were over the esophageal temperature cut-off. According to the Kansas City Classification, in the RFB, group five EDELs were detected (two type 1 lesion and three type 2a lesions), which resolved spontaneously without further sequelae. Complication data are summarized in Table 5.

### 3.4. Outcome Data

**Figure 3:**
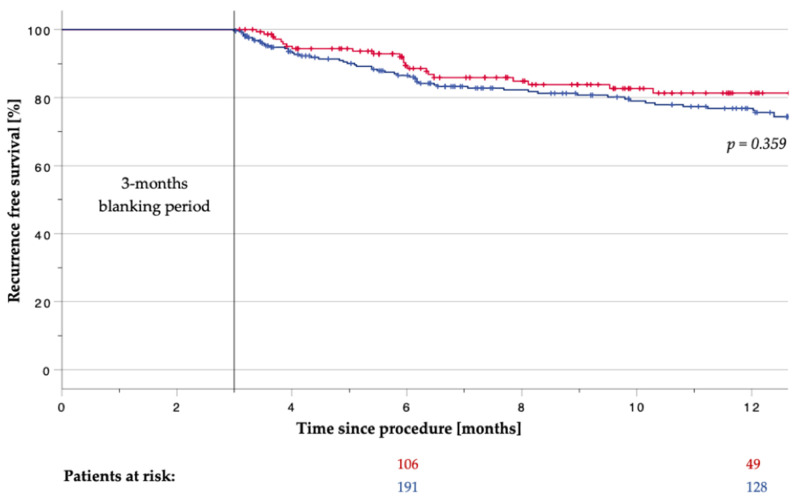
Recurrence-free survival after cryo-balloon (blue) or radiofrequency-balloon (red) pulmonary vein isolation after a 3-month blanking period.

Median follow-up was 292 (172–407) days in the RFB group and 377 (97–816) days in the CB group (*p* = 0.003). A total of 12 patients (7.0%; class III: *n* = 9 (5.3%); class IC: *n* = 3 (1.7%)) were on AAD in the RFB group, and 29 patients (8.5%; class III: *n* = 23 (6.7%); class IC: *n* = 6 (1.8%)) in the CB group, after a 3-month blanking period (*p* = 0.609). Kaplan–Meier estimation showed a recurrence-free survival of 81.3% in the RFB group and 76.8% in the CB group after a 12-month follow-up period (*p* = 0.359, Figure 3).

## 4. Discussion

To the best of our knowledge, this is the first matched analysis comparing the safety, efficacy, and outcome of patients with paroxysmal or persistent AF, treated either with the RFB system or with the CB system.

### 4.1. Efficiency

In this study, AT/AF recurrence-free survival after 12 months is comparable between both groups. The efficiency reported for both systems aligns with the published literature concerning the RFB system (72.2–86.4%) [9,10,11,16] and the CB system (75.3–82.0%) [15,16].

The evaluation of procedural data revealed a significantly longer procedure duration in the RFB group compared to the CB group. The procedure duration of RFB-PVI in our study is similar to the published RFB-PVI procedure duration data, performed under deep sedation [9,10,17,18]. An explanation for the longer procedure duration using the RFB system might be the additional 3D mapping of the left atrium and the more sophisticated balloon preparation. Against expectations, the fluoroscopy time was longer in the RFB group in comparison to the CB group, despite the 3D-mapping integration of the RFB. We hypothesize that additional fluoroscopy was used during the 3D-mapping process of the left atrium, in the sense of an additional procedure step. In addition, the novelty of the RFB and the fact that there is less experience in handling the RFB system might also contribute to the longer procedure duration and fluoroscopy time compared to the established CB system.

The 3D-mapping integration of the RFB enables the generation of a detailed model of the left atrium and can help to respond to special electro-anatomical conditions, e.g., additional or atypical PVs, large common ostia, and left atrial anomalies. In the case of anatomical difficulties, a 3D-tracked segmental or focal ablation with the RFB can be performed, or a total switch to point-by-point RF ablation is possible. Unexpected anatomical anomalies can be challenging for the CB. Furthermore, the visualization of left atrial voltage and location of the ablation line in the 3D model is possible with the RFB. Potentially arrhythmia-inducing substrates can be identified and, if necessary, addressed through focal RF ablation.

With both systems, a high rate of single-shot PVI was possible. However, at the LIPV, the PVI rate with first energy application was lower in the RFB group compared to the CB group. The equatorial location of the RF electrodes requires an accurate alignment of the RFB and PV axis to establish a circular lesion enclosing the PV ostium [11]. This might be one reason for the lower single-shot isolation rate at the LIPV. The CB offers whole-surface energy delivery, enabling maneuvers like a hockey stick to isolate the LIPV, and might cause a higher rate of single-shot isolation. The time-to-isolation at each PV was significantly lower when using the RFB system compared to the CB system. The energy transmission from balloon to tissue using RF energy is faster compared to cryo-energy, in line with the findings of Almorad et al. [16]. The left atrial dwell time was shorter when using the RFB system compared to the CB system, despite the creation of an electroanatomical map of the left atrium. One might hypothesize that the shorter time to isolation at each PV and similar single-shot isolation like in the CB group lead to a shorter left atrial dwell time.

### 4.2. Safety

In this prospective single-center study, no major complications occurred in the RFB group. However, in the CB group, a major complication occurred in four patients (1.2%).

Post-procedure, one CB patient showed motor aphasia. An immediately performed cerebral imaging showed no pathology. All neurologic complaints resolved spontaneously within one hour, leading to the diagnosis of a TIA. Another CB patient showed a drop in blood pressure at the end of the procedure, and echocardiographic examination revealed a pericardial tamponade with the need for immediate peri-cardiocentesis. Hemodynamics stabilized after drainage of the effusion, and there was no need for further surgical treatment.

Although energy delivery was immediately stopped after the weakening of PN capture, two persistent PNP occurred during RSPV ablation in the CB group. Another four CB patients experienced a transient PNP during RSPV ablation, which resolved within 48 h. The PNP rate in the CB group is consistent with the published complication rates when using CB systems [7,8,19]. In the RFB group, only one patient experienced transient PNP. Prior to the ablation at the RSPV, phrenic nerve pacing from the balloon was, for once, not performed [9]. The higher PNP rates in the CB group might be caused by the inability to check phrenic nerve proximity to the CB before ablation. The energy delivery can only be stopped when phrenic nerve capture starts to decline. In contrast, the RFB system allows pacing from the ablation site and repositioning of the balloon before energy delivery in the event of phrenic nerve capture. Remarkably, PNP rates are only lowered using the RFB system if phrenic nerve pacing is performed before ablation of the RSPV [9,10,11,17,18,20]. In summary, phrenic nerve pacing from the balloon’s electrodes seems to be crucial for PNP risk reduction.

The detected difference in vascular access complications can be attributed to the higher rate of sonographic guidance for the femoral venous punctures in the RFB group compared to the CB group. Thus, this difference is not correlated to the PVI modality, in our opinion.

### 4.3. Limitations and Perspectives

Although the present study is a matched analysis, it is not a randomized clinical trial and is therefore limited. Optimizations in the workflow may influence the efficacy and procedural characteristics. No systematic EGD was performed in the CB group.

To acquire additional evidence concerning the comparison of the RFB and the CB, a randomized trial is needed. The value of RFB in the context of re-PVI, substrate ablation, and left atrial flutter ablation should be further investigated.

## 5. Conclusions

Although a longer procedure duration was observed in the RFB group, both technologies enable an efficient and safe PVI. Three-dimensional mapping integration of the RFB may have advantages in comparison to the CB and could provide a more flexible ablation strategy. Regarding fluoroscopy time, the RFB showed no benefit in comparison to the CB.

## Figures and Tables

**Table 1:** Overview of the characteristics of the radiofrequency balloon and the cryo-balloon.

	RF Balloon	Cryo Balloon
Ablation energy	RF	Cryo
Ablation time [s]	60	180 ^1^
3D mapping integration	+	-
Compliant balloon	+	-
Segmental ablation possible	+	-
PN pacing from the balloon possible	+	-

PN, phrenic nerve; RF, radiofrequency; TTI, time to isolation; ‘+’, feature available; ‘-‘, feature not available; ^1^ TTI-guided protocol used.

**Table 2:** Baseline characteristics of the RFB group and the CB group.

Baseline Characteristics	RFB Group *n* = 171	CB Group *n* = 342	*p* Value
Age [years] (mean ± SD)	68.5 ± 10.2	67.9 ± 11.8	0.938
Female, *n* (%)	63 (36.8)	126 (36.8)	1.000
BMI [kg/m^2^] (mean ± SD)	28.5 ± 5.5	28.4 ± 5.2	0.847
Paroxysmal AF, *n* (%)	108 (63.2)	216 (63.2)	1.000
CHA_2_DS_2_-VASc score (mean ± SD)	3.8 ± 2.0	3.6 ± 1.9	0.155
Reduced ejection fraction ^1^, *n* (%)	69 (40.4)	112 (32.7)	0.096
Arterial hypertension, *n* (%)	129 (75.4)	272 (79.5)	0.308
Diabetes mellitus, *n* (%)	35 (20.5)	67 (19.6)	0.907
Prior stroke or TIA, *n* (%)	23 (13.5)	39 (11.4)	0.566
CAD, *n* (%)	91 (53.2)	173 (50.6)	0.649
LAD [mm] (mean ± SD)	45 ± 6	45 ± 6	0.609

AF, atrial fibrillation; BMI, body mass index; CAD, coronary artery disease; CB, cryo-balloon; LAD, left atrial diameter; RFB; radiofrequency balloon; SD, standard deviation; TIA, transient ischemic attack; ^1^ left ventricular ejection fraction < 35%.

**Table 3:** Procedural characteristics of the RFB group and the CB group.

Procedural Characteristics	RFB Group *n* = 171	CB Group *n* = 342	*p* Value
Procedure duration (skin to skin) [min], median (IQR)	88 (70–115)	73 (54–97)	<0.001
Dwell time [min], median (IQR)	23 (15–36)	28 (20–41)	0.006
Fluoroscopy time [min], median (IQR)	18.9 (13.9–29.6)	14.5 (9.8–21.4)	<0.001

CB, cryo-balloon; IQR, interquartile range; RFB, radiofrequency balloon; SD, standard deviation.

**Table 4:** Ablation data of the RFB group and the CB group.

Ablation Data	RFB Group *n* = 171	CB Group *n* = 342	*p* Value
Treated PVs (overall), *n*	669	1355	0.891
LSPV, *n*	161	324	
Single shot isolation, *n* (%)	128 (79.5)	259 (80.2)	0.968
TTI [s] (mean ± SD)	15.6 ± 8.6	47.9 ± 25.0	<0.001
TTI observational rate, *n* (%)	126 (78.3)	268 (82.7)	0.352
energy applications (mean ± SD)	1.7 ± 1.3	1.6 ± 1.1	0.807
LIPV, *n*	161	324	
Single shot isolation, *n* (%)	125 (77.6)	299 (92.6)	<0.001
TTI [s] (mean ± SD)	12.5 ± 6.2	39.4 ± 21.3	<0.001
TTI observational rate, *n* (%)	128 (79.5)	245 (75.6)	0.255
energy applications (mean ± SD)	1.7 ± 1.0	1.4 ± 0.7	0.002
LPV, *n*	8	18	
Single shot isolation, *n* (%)	5 (62.5)	6 (33.3)	- ^2^
TTI [s] (mean ± SD)	11.7 ± 7.2	61.9 ± 37.3	- ^2^
TTI observational rate, *n* (%)	3 (37.5)	12 (66.7)	- ^2^
energy applications (mean ± SD)	3.0 ± 2.1	3.6 ± 2.2	0.629
RSPV, *n*	169	339	
Single shot isolation, *n* (%)	114 (67.5)	216 (63.9)	0.437
TTI [s] (mean ± SD)	10.8 ± 5.4	43.4 ± 24.5	<0.001
TTI observational rate, *n* (%)	133 (78.7)	279 (82.3)	0.355
energy applications (mean ± SD)	1.4 ± 1.1	1.5 ± 0.9	0.306
RIPV, *n*	169	339	
Single shot isolation, *n* (%)	99 (58.6)	203 (60.1)	0.753
TTI [s] (mean ± SD)	11.0 ± 5.0	46.5 ± 25.2	<0.001
TTI observational rate, *n* (%)	130 (76.9)	282 (83.2)	0.109
energy applications (mean ± SD)	1.3 ± 0.6	1.6 ± 1.1	0.005
RMPV, *n*	1	8	
Single shot isolation, *n* (%)	1 (100)	6 (75.0)	- ^2^
TTI [s] (mean ± SD)	35 ^1^	35.5 ± 26.2	- ^2^
TTI observational rate, *n* (%)	1 (100)	2 (25.0)	- ^2^
energy applications (mean ± SD)	1	1.9 ± 0.6	- ^2^
RPV, *n*	0	3	
Single shot isolation, *n* (%)	-	3 (100)	- ^2^
TTI [s]	-	49 ^1^	- ^2^
TTI observational rate, *n* (%)	-	1 (33.3%)	- ^2^
energy applications (mean ± SD)	-	1.7 ± 0.6	- ^2^

CB, cryo-balloon; LIPV, left inferior pulmonary vein; LSPV, left superior pulmonary vein; LPV, left pulmonary veins with common ostium; RFB, radiofrequency balloon; RIPV, right inferior pulmonary vein, RSPV, right superior pulmonary vein; SD, standard deviation; TTI, time to isolation; ^1^ only one TTI was observed—as a consequence, SD calculation is not reasonable; ^2^ not reasonable or not estimable.

**Table 5:** Complication data of the RFB group and the CB group.

Complication Data	RFB Group *n* = 171	CB Group *n* = 342	*p* Value
Major complication, *n* (%)	0 (0)	4 (1.2)	0.307
Fatality	0 (0)	0 (0)	
Pericardial tamponade ^1^, *n* (%)	0 (0)	1 (0.3)	
Stroke or TIA, *n* (%)	0 (0)	1 (0.3)	
Persistent PNP, *n* (%)	0 (0)	2 (0.6)	
Atrio-esophageal fistula, *n* (%)	0 (0)	0 (0)	
Vascular access complication ^1^, *n* (%)	0 (0)	0 (0)	
Minor complication, *n* (%)	6 (3.5)	7 (2.0)	0.374
Pericardial effusion ^2^, *n* (%)	0 (0)	0 (0)	
Transient PNP, *n* (%)	1 (0.6)	4 (1.2)	
Vascular access complication ^2^, *n* (%)	0 (0)	3 (0.9)	
EDEL, *n* (%)	5 (2.9)	NA	

CB, cryo-balloon; EDEL, endoscopically detected esophageal lesion; NA, not applicable; PNP, phrenic nerve palsy; RFB, radiofrequency balloon; ^1^ with the need for specific treatment; ^2^ without need for specific treatment.

## Data Availability

The data presented in this study are available on request from the corresponding author. The data are not publicly available due to data privacy laws.
